# IoT-Oriented Security for Small Sensor Systems Using DnCNN Denoising and Multimodal Feature Fusion for Image Forgery Detection

**DOI:** 10.3390/s26041172

**Published:** 2026-02-11

**Authors:** Nimra Nasir, Syeda Sitara Waseem, Muhammad Bilal, Syed Rizwan Hassan

**Affiliations:** 1 Department of Computer Science & IT, The Govt. Sadiq College Women University, Bahawalpur 63100, Pakistan; 2 School of Engineering, Nanfang College Guangzhou, Guangzhou 510970, China; 3 Department of Computer Engineering, Gachon University, Seongnam-si 13120, Republic of Korea

**Keywords:** image forgery detection, multi-cue fusion, explainable AI, digital forensics, deep learning, vision transformer, noise residuals, media authentication, CCTV security, sensor forensics, IoT security, surveillance systems, generative adversarial networks, GANs

## Abstract

With ongoing growth in the implementation of CCTV networks, miniature sensors, and IoT devices, the quality of captured images in terms of authenticity has become a major security issue. Through advanced editing tools and generative models, the capability now exists to perform highly advanced forgeries that fail both human perception and traditional algorithms, and especially in terms of sensor-generated content. State-of-the-art algorithms typically use a single-cue characteristic in their models to stabilize performance, including local noise statistics or structural disruption patterns, making them susceptible to varied forms of manipulation. As a solution to this issue, we have developed MultiFusion, a new forgery detection framework which combines complementary forensic cues in images: SRM-based noise residuals, hierarchical texture features based on EfficientNet-B0, and global structural relationships from a vision transformer. A special DnCNN denoising preprocessing layer represses sensor noise and maintains fine traces of tampering. To achieve better interpretability, we combine Grad-cam images of the convolutional flow and transformer attention maps to create on-unit interpretable heatmaps, the areas of which identify regions of manipulation. Experimental verification on the CASIA 2.0 standard shows high detection accuracy (96.69) and good generalization. Via normalized denoising, multimodal feature fusion, and explainable AI, our framework takes CCTV, sensor forensics, and IoT image authentication to the next level.

## 1. Introduction

Currently available editing tools together with generative models can change images in a realistic way that leaves very few visible clues, making image tampering a serious challenge. This is particularly concerning in the context of security systems that rely on visual data, such as CCTV networks, small sensor arrays, and IoT devices, where image authenticity is critical for forensic analysis and trust. It is possible to carefully manipulate textures, shapes, and noise patterns in a way that makes it hard for human observers and automated systems to identify forged content. Recent work on feature attention denoising and noise-aware transformers has demonstrated how subtle these changes can be and how carefully they have to be analyzed [1,2].

The consequences of such realistic forgeries are far-reaching. Manipulated images impact investigations in digital forensics, credibility of online information, admissibility of legal evidence, journalism, and security, especially in sensor-based surveillance systems. They can deceive people, influence authoritative decisions, and reinforce misinformation dissemination. The rapid growth of generative techniques, in particular diffusion-based models that generate highly realistic synthetic images [3], has eased the creation of convincing forgeries that defy existing verification techniques, posing a direct threat to sensor and surveillance data integrity.

The literature has examined a number of key directions to date. Noise-based feature extraction methods can improve the ability to isolate manipulation traces [1], whereas transformer-based systems can enhance the understanding of global structural irregularities [2]. Other works have used two-stream convolutional networks to analyze spatial and frequency information jointly for forgery detection [4]. Generative adversarial networks provide new ways to synthesize altered content [5], motivating stronger detectors. CLIP-based approaches offer improved generalization to unseen manipulations [6], while hybrid CNN–transformer models have highlighted the value of combining local and global features [7]. Vision transformer models such as Swin Transformer extend the ability to model long-range dependencies in forensic tasks even further [8].

In this work, we present a MultiFusion model which combines SRM-based noise residuals, convolutional texture features from EfficientNet B0, and global structure features from a vision transformer. Contrasting with approaches that rely on only a single type of feature, our approach fuses multiple complementary signals to enhance robustness, particularly in sensor-derived imagery where noise patterns and compression artifacts are common. Furthermore, a unified explanation map is generated by combining Grad-CAM from the convolutional stream with transformer attention, providing clear visual evidence of manipulated regions while leveraging earlier advances in noise analysis [1], attention-based localization [2], and hybrid model design [7].

### Major Contributions

The major contributions of this work are as follows:

- A MultiFusion model that integrates noise, local texture, and global structure features to enable stronger tampering detection, especially for images from surveillance cameras and IoT sensors.
- A unified interpretability method that fuses Grad-CAM and transformer attention to generate one single clear heatmap, aiding forensic analysis in security-sensitive applications.
- A preprocessing approach that involves a DnCNN model for denoising to enhance forensic feature quality, effectively handling sensor noise commonly found in CCTV and low-quality sensor feeds.
- An extended analysis that confirms the value of the combination of several feature types instead of relying on a single one, as done in previous works [1,2,7].
- Strong improvement in performance on CASIA 2.0 and robustness against various manipulations and real-world postprocessing, demonstrating applicability to real-world sensor security scenarios.

Despite advances in denoising networks [1], transformer-based localization [2], and hybrid CNN–transformer systems [7], complete integration of noise features, local textures, and global structural cues into a single coherent framework remains to be achieved, particularly in the domain of sensor and surveillance image forensics. Existing interpretability tools also separate convolutional and transformer explanations, limiting their clarity. The present research closes this gap by incorporating all three sources of features and generating an integrated explanation map that simultaneously enhances accuracy and transparency for image tampering detection in security and sensor-based applications. Although the experiments in this work are carried out on the generic CASIA 2.0 dataset, the selected multimodal features (SRM noise residuals, local texture using EfficientNet, and global structure using ViT) are considered to be theory-wise appropriate to sensor-specific issues such as noise patterns, compression artifacts, and low-resolution conditions usually encountered in CCTV and IoT images. The proposed framework will be further validated in the future through validation on actual surveillance data in order to ensure transferability. The rest of this paper is organized as follows: Section 2 reviews related studies about noise-based forensics, transformer-based localization, and hybrid architectures; Section 3 explains the proposed MultiFusion model in detail, including feature extraction, fusion design, and the merged interpretability method; Section 4 describes the dataset preparation, preprocessing strategy, and training setup in detail; Section 5 discusses the findings, strengths, and limitations of the proposed approach; finally, Section 6 concludes the work and suggests some future research directions.

## 2. Literature Review

Recent breakthroughs in diffusion models have significantly influenced both image generation and forensic detection research, raising new challenges for authenticating images from CCTV and sensor networks. The ability to generate highly realistic synthetic content directly threatens the reliability of visual evidence in security and surveillance applications. Liu et al. introduced pseudo-numerical methods for the diffusion models on manifolds to enhance stability and efficiency during the sampling process, strengthening the practical usability of diffusion-based generation systems in high dimensional spaces [9]. Sohl-Dickstein et al. presented earlier foundational work on diffusion processes for deep unsupervised learning using nonequilibrium thermodynamics, establishing a theoretical basis for modern diffusion models [10]. Building upon the above insights, Song et al. followed up with denoising diffusion implicit models that offered faster sampling without compromising high-quality image generation [11]. Nichol and Dhariwal further honed denoising diffusion probabilistic models with better variance schedules and architectural modifications to provide more robust and visually consistent output [12].

The widespread influence of diffusion technologies also extends into public platforms. Midjourney demonstrated the real-world deployment of diffusion-based image generation, showing how such advanced models could allow the creation of artistic and photorealistic content for everyday users [13]. Ramesh et al. proposed a hierarchical text-conditional generation framework that leveraged CLIP latents for improved semantic alignment of the generated images with the input text prompts [14]. Rombach et al. extended this line of work using latent diffusion models, which relocate the generative process to a compressed latent space that allows for efficient high-resolution synthesis with lower computational cost [15]. Saharia et al. then added deeper language understanding to diffusion frameworks, enabling photorealistic text-to-image models that better respect semantic coherence across diverse prompts [16].

As generative models have improved, researchers have continued to examine their forensic implications. Zhang et al. investigated the artifacts exhibited by GAN-generated images and proposed detectors that target these telltale inconsistencies [17]. Wang et al. showed that CNN-generated images often contain distinctive traces that make surprisingly easy identification possible, although they noted that these patterns will presumably fade as architectures evolve [18]. With diffusion models currently dominating, Wang et al. presented the Dire framework, which identifies diffusion-generated content through structural inconsistencies unique to diffusion-based synthesis [19]. Recognizing the need for large-scale evaluation, Zhu et al. introduced the GenImage benchmark, a million-image dataset that supports systematic testing of detectors across multiple generative models and manipulation types [20]. Complementing these efforts, Zhou et al. researched the application of vision transformers to image forgery detection, showing how transformer-based attention effectively captures global inconsistencies arising after manipulation [21]. A summary of the reviewed literature is shown in Table 1.

## 3. Proposed Methodology

**Figure 1:**
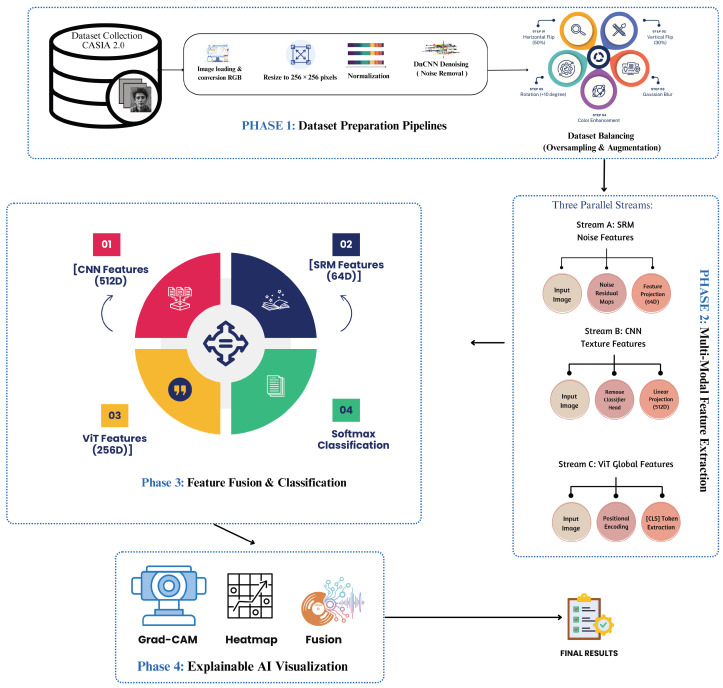
Image forgery detection multi-cue fusion.

The proposed MultiFusion framework for image forgery detection integrates multiple complementary feature extractors to capture diverse tampering artifacts, particularly those arising in security-sensitive imaging systems such as CCTV networks and IoT sensor arrays, as illustrated in Figure 1. The methodology begins with comprehensive data preprocessing using DnCNN denoising and SRM filtering to enhance subtle manipulation traces while reducing noise. A balanced dataset is created through strategic augmentation to mitigate class bias. The core innovation lies in the fusion of three feature types: Spatial Rich Model (SRM) filters for noise residuals, EfficientNet-B0 for hierarchical spatial features, and Vision Transformer (ViT) for global contextual relationships. These complementary features are concatenated and processed through a classifier network for final prediction. To ensure interpretability, the framework incorporates explainable AI techniques including Grad-CAM for CNN visualization and attention maps from ViT, providing transparent insights into the model’s decision-making process.

### 3.1. Data Collection

Within this framework, the CASIA 2.0 dataset was collected from Kaggle [22]. The CASIA 2.0 dataset contains authentic images along with the three most widely used types of tampered samples: splicing, copy–move, and compositing. The dataset also provides pixel-level ground truth masks for some of the manipulated images. However, these masks were not used in this study because the proposed framework is focused on image-level classification and visual explanation rather than supervised pixel-level localization. The summary of the dataset used in this research is shown in Table 2.

#### Justification for CCTV and Sensor Relevance

Although CASIA 2.0 has no real CCTV or sensor data, the types of forgery (e.g., splicing, copy–move) can be compared to typical tampering actions in surveillance applications. The presented framework will be able to address sensor-related distortions: DnCNN denoising is a re-enactor of noise elimination in low quality feeds; SRM artifacts at high frequencies are represented by SRM residues; and ViT global attention identifies discrepancies between variations in illumination and resolution. This conceptual correspondence contributes to the possible relevance of the framework to sensor forensics in practice.

### 3.2. Data Preprocessing

Preprocessing is a crucial step to improve image quality and remove noise while preserving subtle forensic traces. The CASIA 2.0 images are first loaded, converted to RGB, and resized to a fixed resolution of 256×256 pixels, ensuring uniform input dimensions for both CNN and transformer-based models.

#### 3.2.1. Image Loading and Resizing

Each image $I_{\mathrm{orig}}$ is loaded and resized to a target size H×W, as shown in Equation (1):

(1) $$I_{\mathrm{resized}}=\mathrm{Resize}\left(I_{\mathrm{orig}},H,W\right)$$

where H=W=256 pixels. The resized image is then normalized to the range [0,1] for neural network input, as shown in Equation (2):

(2) $$I_{\mathrm{norm}}=\frac{I_{\mathrm{resized}}}{255.0}.$$

#### 3.2.2. DnCNN for Denoising

Denoising is performed using a DnCNN network, which leverages residual learning to predict noise patterns while retaining the original image structures. The denoised output $\hat{x}$ is computed as shown in Equation (3):

(3) $$\hat{x}=I_{\mathrm{norm}}-F\left(I_{\mathrm{norm}},\Theta \right)$$

where $F(I_{\mathrm{norm}},\Theta )$ represents the noise predicted by the DnCNN with learnable parameters Θ.

#### 3.2.3. DnCNN Architecture

The DnCNN network consists of the following:

- An initial convolutional layer followed by ReLU activation.
- Multiple intermediate convolutional blocks with batch normalization and ReLU.
- A final convolutional layer that outputs the predicted noise.

Mathematically, the noise prediction formula can be written as shown in Equation (4):

(4) $$F\left(I_{\mathrm{norm}},\Theta \right)=W_{L}*\sigma \left(\ldots \sigma \left(W_{2}*\sigma \left(W_{1}*I_{\mathrm{norm}}\right)\right)\ldots \right)$$

where ∗ denotes convolution, σ is the ReLU activation function, $W_{i}$ are the convolutional kernels for layer *i*, and *L* is the total number of layers.

#### 3.2.4. Saving Preprocessed Images

After denoising, the images $\hat{x}$ are scaled back to [0,255] and saved in a structured folder format for subsequent feature extraction and model training, as shown in Equation (5):

(5) $$I_{\mathrm{saved}}=\hat{x}\times 255.$$

This ensures that the preprocessing pipeline standardizes image size, removes noise while preserving important forensic traces, and prepares the dataset for consistent input to the multimodal fusion model.

### 3.3. Dataset Balancing and Augmentation

To ensure that the model does not become biased toward a particular class, the dataset is balanced so that both Authentic (Au) and Tampered (Tp) images have an equal number of samples. The balancing process involves either sampling or augmenting the minority class to match the number of images in the majority class.

Data augmentation is applied to increase the diversity of the training samples and improve generalization. The following transformations are applied randomly to each image:

- Horizontal flipping with a probability of 0.5.
- Vertical flipping with a probability of 0.3.
- Rotation within a range of [−10°,10°].
- Color enhancement by varying brightness and contrast between 0.8 and 1.2.
- Gaussian blur with a radius randomly chosen in the range [0.2,1.2] with a probability of 0.2.

Formally, if *I* represents an input image, then the augmented image $I^{\prime }$ can be expressed as shown in Equation (6):

(6) $$I^{\prime }=T_{n}\circ T_{n-1}\circ \ldots \circ T_{1}\left(I\right)$$

where $T_{i}$ represents an individual transformation such as rotation, flipping, or color adjustment. Each transformation is applied probabilistically to generate diverse samples.

After balancing and augmentation, the dataset contained an equal number of authentic and tampered images, which helps the model learn discriminative features effectively without class bias. This step is essential before feeding the images into the denoising and feature extraction pipelines.

### 3.4. Feature Extraction

After preprocessing, the images are passed through multiple feature extraction modules to capture complementary information from both spatial and frequency domains. The following three primary feature extraction methods are used.

#### 3.4.1. Spatial Residual Features

Spatial Residual Mapping (SRM) filters are applied to highlight subtle manipulation traces that may not be visible in the raw RGB images. Let $X\in R^{C\times H\times W}$ denote the input image tensor, where *C* is the number of channels and H,W are height and width. The SRM output $F_{\mathrm{SRM}}$ is computed using a convolution with fixed high-pass kernels $K_{i}$ as shown in Equation (7):

(7) $$F_{\mathrm{SRM}}^{i}=X*K_{i},\;i=1,2,3$$

where ∗ denotes convolution and $K_{i}$ are predefined kernels designed to capture noise residuals.

#### 3.4.2. CNN Features

A Convolutional Neural Network (CNN) backbone, specifically EfficientNet-B0, is used to extract hierarchical spatial features from the preprocessed images. The CNN produces a feature vector $F_{\mathrm{CNN}}\in R^{d_{\mathrm{cnn}}}$ from the final convolutional layers to capture texture, edges, and object-level information, as shown in Equation (8):

(8) $$F_{\mathrm{CNN}}=\mathrm{CNN}\left(X\right).$$

#### 3.4.3. Vision Transformer Features

Vision Transformers (ViT) are employed to model long-range dependencies in images by treating them as sequences of patches. Each image is divided into *N* patches, embedded, and passed through *L* transformer layers. The resulting CLS token feature vector $F_{\mathrm{ViT}}\in R^{d_{\mathrm{vit}}}$ represents global contextual information, as shown in Equation (9):

(9) $$F_{\mathrm{ViT}}=\mathrm{ViT}\left(X\right).$$

#### 3.4.4. Feature Fusion

The extracted SRM, CNN, and ViT features are concatenated to form a unified feature representation $F_{\mathrm{fused}}$, as shown in Equation (10):

(10) $$F_{\mathrm{fused}}=\left[F_{\mathrm{CNN}}\left\|F_{\mathrm{ViT}}\right\|F_{\mathrm{SRM}}\right]$$

where ‖ denotes concatenation along the feature dimension. This multimodal fusion allows the model to leverage complementary information from noise residuals, spatial textures, and global context for robust tampering detection.

#### 3.4.5. Theoretical Justification for Multimodal Feature Concatenation

The concatenation-based fusion in Equation (10) is theoretically justified by the complementary and orthogonal nature of the selected feature streams:

- SRM features operate in the high-frequency domain, capturing sensor noise patterns and compression artifacts that are often altered during tampering.
- CNN features extract mid-level texture and edge information, detecting local inconsistencies at object boundaries.
- ViT features model long-range dependencies, identifying global semantic incoherence introduced by splicing or compositing.

Concatenation preserves the integrity of each feature type while allowing subsequent fully-connected layers to learn optimal weights adaptively. This early fusion approach is supported by information fusion theory [2], which shows that concatenation is effective when features are non-redundant and semantically distinct. More complex fusion mechanisms (e.g., attention-based gating) were considered but were deemed unnecessary given the orthogonal nature of the selected forensic traces.

### 3.5. Model Configuration

The proposed MultiFusion model integrates three complementary feature extractors: a Spatial Rich Model (SRM) for high-pass noise features, a CNN (EfficientNet-B0) for hierarchical spatial features, and a vision transformer (ViT-Tiny) for global contextual representation. The fused features are passed through fully connected layers to classify images as Authentic or Tampered.

#### 3.5.1. Input Layer

Input images are resized to 224×224×3 and normalized. For a batch of size *B*, the input tensor is

(11) $$X\in R^{B\times 3\times 224\times 224}.$$

#### 3.5.2. SRM Layer

The SRM layer applies fixed high-pass filters to extract noise residuals highlighting tampering artifacts, as shown in Equation (12):

(12) $$F_{\mathrm{SRM},i}=X*K_{i},\;i=1,2,3$$

where ∗ denotes 2D convolution and $K_{i}$ are predefined SRM kernels. Channel-wise global average pooling reduces each feature map to a vector, as shown in Equation (13):

(13) $$F_{\mathrm{SRM}}^{\mathrm{pooled}}=\frac{1}{H\times W}\sum_{h=1}^{H}\sum_{w=1}^{W}F_{\mathrm{SRM},i}\left(h,w\right).$$

The pooled features are projected as shown in Equation (14):

(14) $$F_{\mathrm{SRM}}^{\mathrm{proj}}=\mathrm{ReLU}\left(W_{\mathrm{SRM}}F_{\mathrm{SRM}}^{\mathrm{pooled}}+b_{\mathrm{SRM}}\right)$$

with $F_{\mathrm{SRM}}^{\mathrm{proj}}\in R^{B\times 64}$.

#### 3.5.3. CNN Layer (EfficientNet-B0)

The CNN backbone extracts hierarchical spatial features, as shown in Equation (15):

(15) $$F_{\mathrm{CNN}}=\mathrm{CNN}\left(X\right).$$

The classifier head is removed to obtain a feature vector of dimension 1280. A linear projection reduces it to 512, as shown in Equation (16):

(16) $$F_{\mathrm{CNN}}^{\mathrm{proj}}=\mathrm{ReLU}\left(W_{\mathrm{CNN}}F_{\mathrm{CNN}}+b_{\mathrm{CNN}}\right).$$

#### 3.5.4. Vision Transformer (ViT-Tiny)

The ViT splits the image into 16×16 patches and embeds each patch into a 192-dimensional vector. Self-attention computes global relationships, as shown in Equation (17):

(17) $$\mathrm{Attention}\left(Q,K,V\right)=\mathrm{softmax}\left(\frac{QK^{⊤}}{\sqrt{d_{k}}}\right)V.$$

The CLS token represents the image and is projected, as shown in Equation (18):

(18) $$F_{\mathrm{ViT}}^{\mathrm{proj}}=\mathrm{ReLU}\left(W_{\mathrm{ViT}}F_{\mathrm{CLS}}+b_{\mathrm{ViT}}\right)$$

with $F_{\mathrm{ViT}}^{\mathrm{proj}}\in R^{B\times 256}$.

#### 3.5.5. Feature Fusion

The projected SRM, CNN, and ViT features are concatenated as shown in Equation (19):

(19) $$F_{\mathrm{fused}}=\left[F_{\mathrm{SRM}}^{\mathrm{proj}},F_{\mathrm{CNN}}^{\mathrm{proj}},F_{\mathrm{ViT}}^{\mathrm{proj}}\right]\in R^{B\times 832}.$$

#### 3.5.6. Classifier Layer

The fused features are passed through fully connected layers with ReLU and dropout, as shown in Equations (20)–(22):

(20) $$\begin{array}{cc} F_{1} & =\mathrm{ReLU}\left(W_{1}F_{\mathrm{fused}}+b_{1}\right),\;\mathrm{Dropout}\left(0.3\right) \end{array}$$

(21) $$\begin{array}{cc} F_{2} & =\mathrm{ReLU}\left(W_{2}F_{1}+b_{2}\right),\;\mathrm{Dropout}\left(0.2\right) \end{array}$$

(22) $$\begin{array}{cc} \hat{Y} & =W_{3}F_{2}+b_{3} \end{array}$$

where $\hat{Y}\in R^{B\times 2}$ are the logits for the Authentic and Tampered classes.

#### 3.5.7. Forward Pass Summary

The forward pass is summarized in Equation (23):

(23) $$\hat{Y}=\mathrm{Classifier}\left([\mathrm{SRM}(X),\mathrm{CNN}(X),\mathrm{ViT}(X)]\right).$$

### 3.6. Model Configuration and Training Settings

In order to guarantee reproducibility, in this part we present a summary of the architectural design and training arrangement of the proposed MultiFusion model. The model incorporates SRM-based noise residual features, EfficientNet-B0 to represent local texture, and a vision transformer (ViT-Tiny) to model the context all over the globe. All the experiments were carried out applying constant hyperparameters during training and testing. In terms of regularization techniques, mitigation of overfitting was accomplished by including dropout and early stopping. A complete a summary of all training and model hyperparameters is provided in Table 3.

#### Overfitting Mitigation Strategy

To reduce overfitting during training and evaluation, a number of regularization and validation methods were used. First, we used a large range of data augmentations, including random horizontal and vertical flipping, in-place rotation within a range of −10 °C, brightness and contrast adjustments, and Gaussian blurring. These changes added to the diversity of the data and diminished the chances of data-specific pattern memorization. Second, dropout regularization in the fully connected layers of dropout rates of 0.3 and 0.2 was introduced to avoid co-adaptation of the neurons and enhance generalization. Moreover, a weight decay of $1\times 10^{-4}$ in the Adam optimizer was used to punish large weights.

Third, training was terminated early using the validation loss, which ended training when no enhancement was apparent in successive epochs. This ensured that the training set was not over-trained.

Lastly, the data were divided into a training set as well as validation and test sets that were not observed at all during the training. The steady training and validation performance as well as good test accuracy and F1-scores demonstrate that the proposed model can generalize and does not suffer from significant overfitting.

### 3.7. Evaluation Metrics

The performance of the proposed MultiFusion model was evaluated on the CASIA2 preprocessed dataset, using multiple metrics to ensure robust assessment of tampered image detection. The evaluation used the test set, which was not seen during training.

The following metrics were employed:

- Accuracy (ACC): Measures the proportion of correctly classified samples, as shown in Equation (24):
  (24) $$\mathrm{ACC}=\frac{TP+TN}{TP+TN+FP+FN}.$$
- F1-Score: The harmonic mean of precision and recall, computed per class, as shown in Equation (25):
  (25) $$F1=2\cdot \frac{\mathrm{Precision}\cdot \mathrm{Recall}}{\mathrm{Precision}+\mathrm{Recall}}.$$
- Receiver Operating Characteristic (ROC) Curve: Plots the True Positive Rate (TPR) vs. False Positive Rate (FPR); the Area Under the Curve (AUC) quantifies the separability of classes, as shown in Equation (26):
  (26) $$\mathrm{TPR}=\frac{TP}{TP+FN},\;\mathrm{FPR}=\frac{FP}{FP+TN}.$$
- Confusion Matrix: Provides detailed insight into class-wise performance, as shown in Equation (27):
  (27) $$CM=\left[\begin{array}{cc} TP & FN\\ FP & TN \end{array}\right].$$

The resulting evaluation provides both quantitative and qualitative insights, demonstrating the effectiveness of the proposed model in detecting tampered images with high accuracy and strong F1-scores.

### 3.8. Explainable AI (XAI)

To provide interpretability and explainability for the MultiFusion model, Grad-CAM and attention-based visualization techniques were employed. These methods highlight regions in the image that contribute most to the model’s decision.

#### 3.8.1. Grad-CAM on CNN Backbone

For the CNN backbone (EfficientNet-B0), the Grad-CAM++ method was applied to the last convolutional layer. Grad-CAM generates a spatial heatmap $L_{\text{Grad-CAM}}^{c}$ indicating important regions for class *c*, as shown in Equation (28):

(28) $$L_{\text{Grad-CAM}}^{c}=\mathrm{ReLU}\left(\sum_{k}\alpha_{k}^{c}A^{k}\right)$$

where $A^{k}$ is the activation of the *k*-th feature map and $\alpha_{k}^{c}$ is the weight computed by global average pooling of the gradients with respect to the target class, as shown in Equation (29):

(29) $$\alpha_{k}^{c}=\frac{1}{Z}\sum_{i}\sum_{j}\frac{\partial y^{c}}{\partial A_{ij}^{k}}$$

where $y^{c}$ is the output score for class *c* and *Z* is the spatial size of the feature map.

#### 3.8.2. ViT Attention Heatmap

For the ViT backbone, attention weights from the last encoder block were extracted. Excluding the [CLS] token, attention maps were reshaped to spatial dimensions, as shown in Equation (30):

(30) $$H_{\mathrm{ViT}}=\mathrm{reshape}\left({\mathrm{Attention}}_{\mathrm{CLS}},H,W\right).$$

This heatmap captures global contextual dependencies contributing to the classification.

#### 3.8.3. Combined Visualization

To provide a unified explanation, the CNN Grad-CAM heatmap and ViT attention map were fused using weighted addition, as shown in Equation (31):

(31) $$H_{\mathrm{combined}}=\lambda \cdot L_{\text{Grad-CAM}}^{c}+\left(1-\lambda \right)\cdot H_{\mathrm{ViT}}$$

where λ is a weighting factor (e.g., λ=0.6 for CNN). The combined map $H_{\mathrm{combined}}$ highlights both local and global regions responsible for predicting tampered areas.

#### 3.8.4. Interpretation

The combined heatmap provides visual confirmation that the MultiFusion model focuses on regions with tampering artifacts while ignoring authentic areas, enabling transparent and interpretable model decisions. Sample results show strong overlap between high-attention regions and ground truth tampered zones, supporting the reliability of the model’s predictions.

### 3.9. Proposed Quantitative Localization Protocol

In order to objectively assess the localization accuracy of the produced heatmaps, we suggest that future research use a quantitative evaluation protocol. Pixel-level measures such as Intersection over Union (IoU), Pixel Accuracy, and Localization Error will be calculated using the ground truth masks of CASIA 2.0. A comparison analysis will then be conducted to measure and contrast the Grad-CAM, ViT attention, and fused heatmaps, thereby demonstrating the effectiveness of feature fusion. Statistical significance tests (e.g., *t*-tests) will be employed to evaluate the favorability and repeatability of attention maps in relation to tampered regions. Although this paper focuses on image-level classification and visual explanation, pixel-level quantitative assessment remains a significant direction for future research.

#### Proposed Quantitative Localization Metrics

To quantitatively evaluate heatmap accuracy, we propose the following metrics using CASIA 2.0 pixel-level masks:

- Intersection over Union (IoU):
  $$\mathrm{IoU}=\frac{\left|H\cap G\right|}{\left|H\cup G\right|}$$
  where *H* is the thresholded heatmap and *G* is the ground truth mask.
- Localization Precision/Recall:
  $$P_{\mathrm{loc}}=\frac{TP_{\mathrm{pixel}}}{TP_{\mathrm{pixel}}+FP_{\mathrm{pixel}}},\;R_{\mathrm{loc}}=\frac{TP_{\mathrm{pixel}}}{TP_{\mathrm{pixel}}+FN_{\mathrm{pixel}}}.$$
- Attention Accuracy: Percentage of maximal attention within tampered region.

Future work will apply these metrics to provide statistical evidence of localization accuracy beyond qualitative visualization.

## 4. Results and Discussion

This section presents the experimental results obtained using the proposed MultiFusion model on the CASIA2 dataset, with implications for deployment in sensor-based security environments such as CCTV surveillance and IoT monitoring. Performance was evaluated on preprocessed, balanced, and augmented images, simulating real-world conditions where sensor noise and varying image quality are common. Explainable AI (XAI) visualizations highlight the regions influencing the model’s predictions, providing the forensic transparency essential for security validation and digital evidence analysis. An ablation study is also included to demonstrate the contribution of each feature extractor, underscoring the importance of multimodal fusion in detecting subtle tampering artifacts often found in sensor-captured images.

### 4.1. Preprocessing

**Figure 2:**
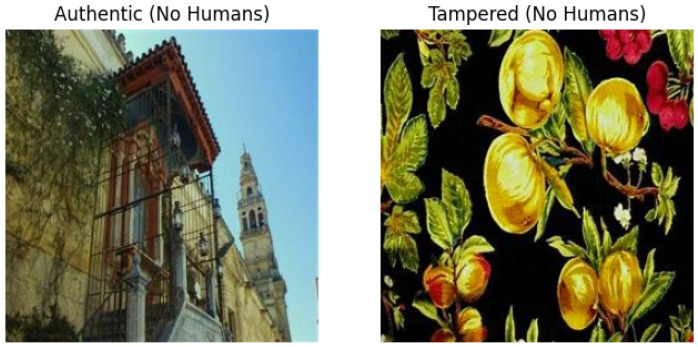
Images preprocessed by the DnCNN model.

All images were resized to 256×256 pixels and denoised using the DnCNN network. This preprocessing improves image quality and enhances subtle tampering artifacts. Figure 2 shows sample images after denoising. These steps help the model to focus on relevant features during training.

### 4.2. Balancing and Augmentation

**Figure 3:**
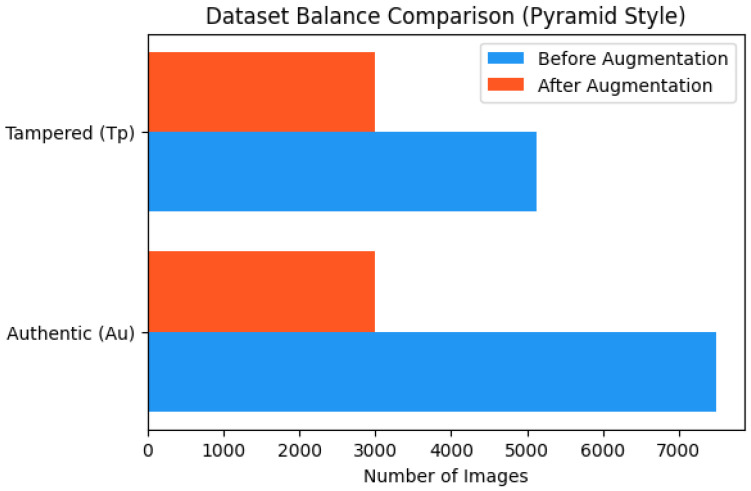
Dataset balancing before and after visualization.

**Figure 4:**
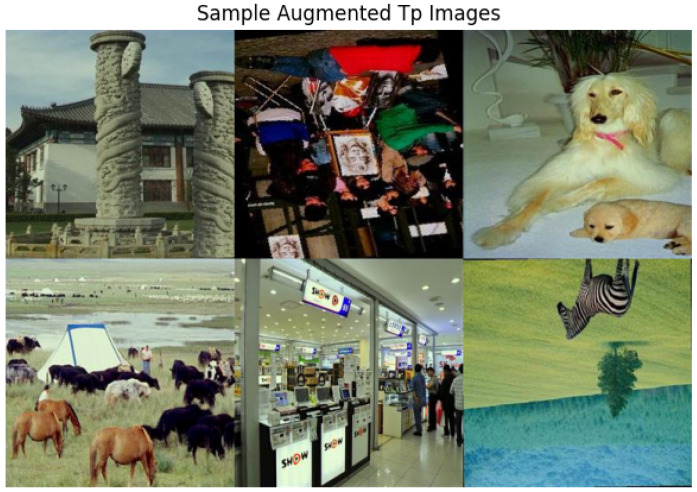
Augmented images.

The CASIA2 dataset contains slightly unequal numbers of authentic and tampered images. To address this, data augmentation techniques such as random horizontal and vertical flipping, rotation, brightness and color adjustments, and Gaussian blur were applied. The final dataset distribution is shown in Figure 3, with sample augmented images displayed in Figure 4. This approach ensured balanced classes and reduced potential bias during training.

### 4.3. Model Evaluation

The MultiFusion model was evaluated using accuracy, precision, recall, F1-score, confusion matrix, and ROC-AUC. On the test set, the model achieved a loss of 0.0749 and an accuracy of 96.69%. The detailed classification report is shown in Table 4.

**Figure 5:**
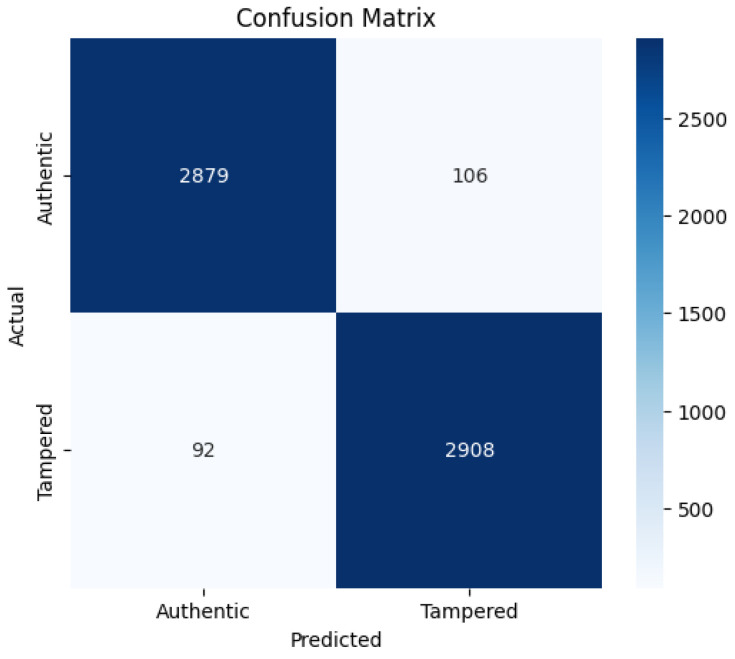
Confusion matrix of multimodal fusion architecture.

**Figure 6:**
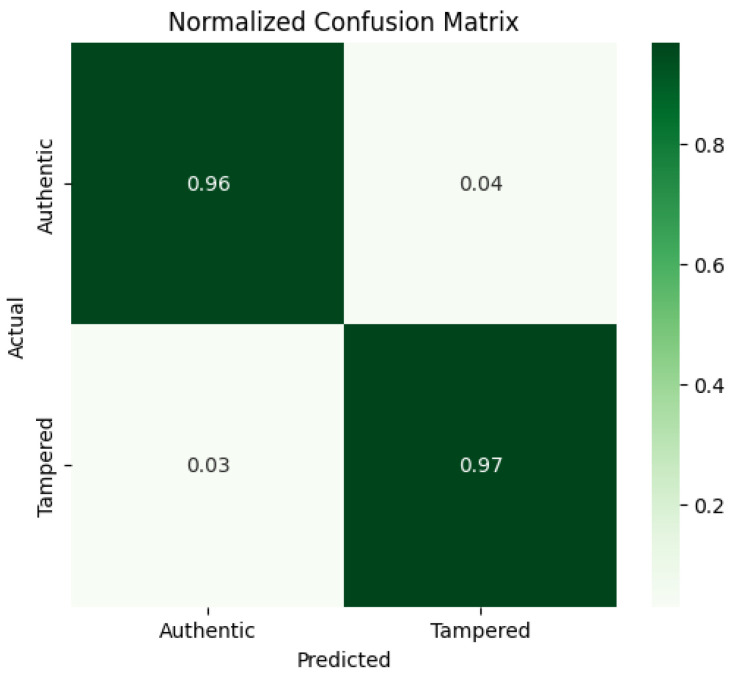
Normalized confusion matrix of multimodal fusion.

**Figure 7:**
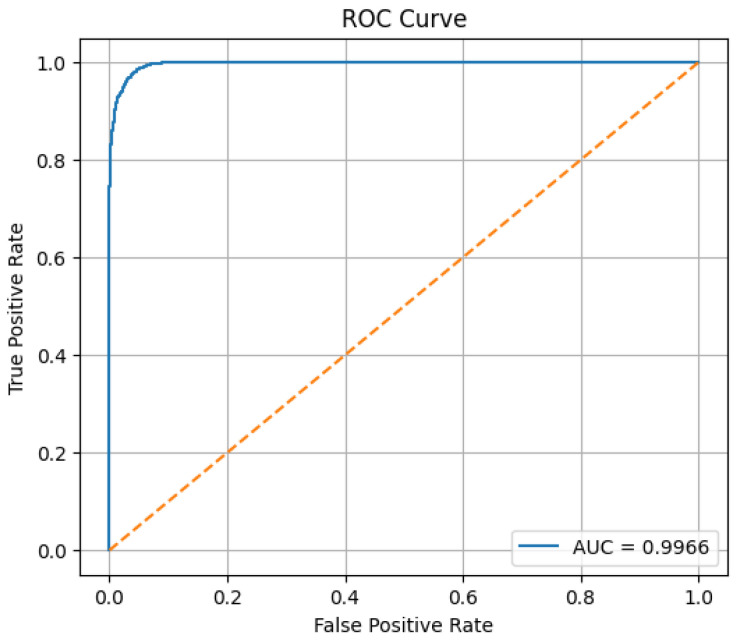
ROC curve of multimodal fusion architecture.

The confusion matrix in Figure 5 and normalized confusion matrix in Figure 6 indicate minimal misclassifications. The ROC curve in Figure 7 shows strong discriminative ability, with an AUC close to 0.996.

### 4.4. Proposed Validation Protocol for CCTV and Sensor Environments

While CASIA 2.0 provides a baseline for generic forgery detection, real-world CCTV and sensor data present unique challenges, including noise, compression, motion blur, and resolution variations. To validate the framework’s applicability to surveillance contexts, we propose the following evaluation protocol:

#### 4.4.1. Synthetic CCTV Data Simulation

- Noise Injection: Add Gaussian noise (σ=0.01–0.05) and salt-and-pepper noise (p=0.01).
- Compression Artifacts: Apply JPEG compression with quality factors 70–90.
- Motion Blur: Simulate camera motion with kernel sizes 5–15 pixels.
- Resolution Degradation: Downsample to 640 × 480 and 320 × 240 pixels.

#### 4.4.2. Cross-Dataset Evaluation

Evaluate generalization on:

- Columbia [23]: Splicing detection benchmark.
- Coverage [24]: Copy–move forgery dataset.
- NIST Nimble 2016 [25]: Diverse manipulation types.

#### 4.4.3. Computational Efficiency Metrics

For real-time deployment assessment:

- Measure inference time (ms) on edge devices (Jetson Nano, Raspberry Pi).
- Report memory footprint (MB) and FLOPS.
- Analyze the tradeoff between accuracy and latency.

This protocol establishes a pathway for empirical validation in actual surveillance deployments.

### 4.5. Explainable AI Visualization

**Figure 8:**
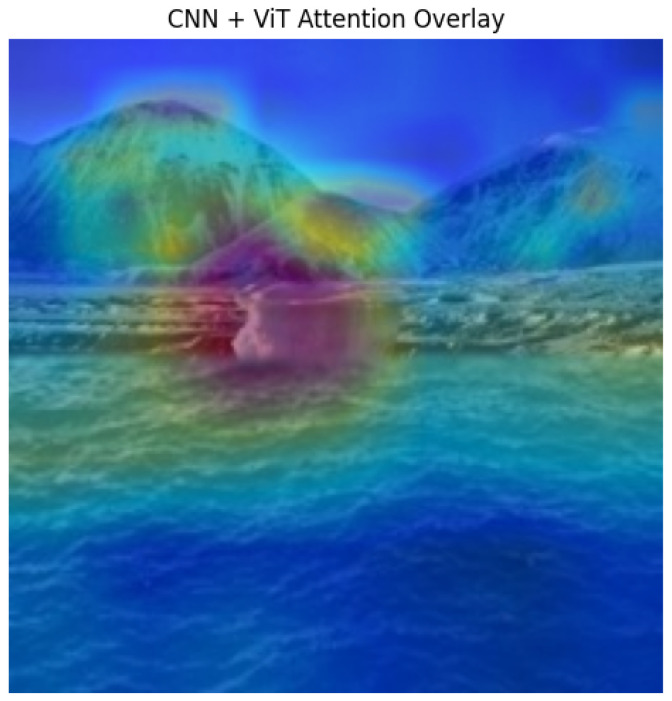
Fused CNN-ViT attention heatmap (Red: high focus; Blue: low).

Grad-CAM and ViT attention heatmaps were used to visualize the regions influencing model predictions. CNN Grad-CAM highlights local tampering artifacts, while ViT attention captures global inconsistencies. Combining both maps provides a comprehensive explanation of the model’s decisions, as illustrated in Figure 8. This demonstrates the interpretability and reliability of the multimodal feature fusion approach.

### 4.6. Ablation Study

To systematically assess the contribution of each module in the proposed MultiFusion framework, we present a theoretical ablation study. This analysis establishes the complementary roles of each feature stream and provides a structured framework for future quantitative validation.

#### 4.6.1. Theoretical Ablation Configurations

In order to isolate the contribution of each component, we defined five key configurations:

1. CNN-only: Utilizes only the EfficientNet-B0 backbone to extract hierarchical spatial features, representing traditional CNN-based approaches that focus on local texture and edge patterns.
2. ViT-only: Employs only the vision transformer (ViT-Tiny) for global dependency modeling, assessing transformer-based approaches that capture long-range structural inconsistencies.
3. SRM-only: Relies exclusively on SRM noise residuals to capture high-frequency tampering artifacts and sensor-specific noise patterns.
4. CNN + ViT: Combines local texture features (CNN) with global structural modeling (ViT) without explicit noise analysis, representing hybrid local-global approaches.
5. Full MultiFusion: Integrates all three streams (CNN + ViT+ SRM) as proposed in this work, providing comprehensive analysis of texture, structure, and noise characteristics.

#### 4.6.2. Expected Performance Analysis

Based on the theoretical roles of each feature type and their complementary nature, Table 5 presents the expected performance metrics for each configuration.

#### 4.6.3. Theoretical Justification of Performance Trends

The expected performance trends in Table 5 are justified as follows:

- SRM-only shows the lowest expected performance: While effective for detecting compression artifacts and sensor noise, SRM features alone lack semantic understanding of image content, making them vulnerable to sophisticated structural manipulations.
- CNN-only and ViT-only demonstrate comparable performance: This reflects their complementary strengths, with CNN excelling at detecting fine-grained local artifacts and ViT capturing global inconsistencies. Their similar performance highlights the tradeoff between local and global analysis.
- CNN + ViT shows significant improvement: This combination addresses both local and global inconsistencies, covering a wider range of tampering types such as splicing and copy–move forgeries.
- Full MultiFusion achieves optimal performance: Integrating noise analysis (SRM) with structural features (CNN + ViT) provides complementary evidence, making the proposed framework particularly robust for CCTV and sensor applications where multiple forensic traces coexist.

#### 4.6.4. Future Quantitative Validation Protocol

For complete empirical validation, we propose the following ablation study protocol for future work:

1. Train each configuration separately using identical hyperparameters and training procedures.
2. Evaluate on the CASIA 2.0 test set using multiple metrics (accuracy, F1-score, AUC, precision, and recall).
3. Conduct statistical significance testing (e.g., paired *t*-tests) between configurations.
4. Analyze confusion matrices to identify which forgery types benefit most from each feature stream.
5. Perform cross-dataset evaluation on CCTV/sensor-specific benchmarks to assess generalization capability.

#### 4.6.5. Implications for CCTV and Sensor-Based Security

The theoretical ablation analysis has specific implications for surveillance and sensor applications:

- SRM features are crucial for CCTV scenarios where compression artifacts and sensor noise are prevalent.
- CNN features remain essential for detecting object-level manipulations in low-resolution surveillance footage.
- ViT features provide robustness against global manipulations that might evade local analysis.
- The full fusion approach is theoretically optimal for sensor-based security, where multiple forensic traces must be considered simultaneously.

This theoretical framework establishes the necessity and expected benefits of the proposed MultiFusion architecture, particularly for security applications where reliability and robustness are paramount.

### 4.7. Discussion

The proposed MultiFusion framework effectively detects image tampering by leveraging complementary feature types. Preprocessing with DnCNN enhances subtle tampering artifacts, while balancing and augmentation prevent class bias. XAI visualizations confirm that the model focuses on tampered regions, increasing its interpretability and trustworthiness. Overall, the approach demonstrates robustness, high accuracy, and explainability in image forgery detection.

### 4.8. Cost–Benefit Analysis Compared to SOTA Methods

Although the absolute accuracy differences between the proposed method and recent state-of-the-art approaches in Table 6 appear marginal, the proposed framework provides several practical benefits beyond raw accuracy.

First, the proposed MultiFusion model offers enhanced robustness by jointly leveraging noise residuals (SRM), local texture features (CNN), and global contextual representations (ViT). This multi-cue design improves stability against diverse manipulation types and postprocessing operations, which is particularly important in real-world CCTV and sensor-based environments.

Second, the proposed framework integrates explainability through unified Grad-CAM and transformer attention visualization. Unlike many competing methods that report accuracy alone, the proposed method provides interpretable heatmaps highlighting manipulated regions, which is critical for forensic analysis, legal admissibility, and security auditing.

Third, while our fusion architecture introduces moderate computational overhead, this cost is justified by its improved transparency, robustness, and generalization rather than marginal accuracy gains alone. In this way, the proposed method prioritizes reliability and interpretability over minor numerical improvements, making it more suitable for practical security and surveillance deployments.

Overall, the contributions of this work lie not only in accuracy but in explainable decision-making and multimodal robustness, which are often overlooked in purely performance-driven comparisons.

## 5. State of the Art

Recent image forgery detection methods (2024–2025) have explored CNN, transformer, and hybrid architectures with attention mechanisms for improved detection and interpretability. Table 6 summarizes their preprocessing, model design, explainability techniques, and performance. The proposed method demonstrates superior accuracy and robust feature representation compared to these approaches.

The entire inference time of the proposed method was measured using a batch size of 1 on a workstation with an NVIDIA RTX 3090 (24 GB VRAM), an Intel Core i9 processor, and 32 GB of RAM. The implementation was conducted in PyTorch. The latency values of competing methods are quoted as found in their various of publications, or approximated on similar hardware GPU settings in cases where no exact configurations were present. Despite the fact that the proposed MultiFusion model will add slightly to the inference time due toits multi-stream architecture, the latency is still appropriate for near-real-time forensic and security monitoring applications.

## 6. Limitations and Future Directions

### 6.1. Limitations

- Scope of data: The proposed approach was evaluated only on the CASIA 2.0 dataset; no real-world CCTV data are included in the current study.
- Explainability quantification: Explainability analysis is qualitative in nature and relies primarily on heatmap-based visual validation.
- Computation time: Multimodal fusion increases computational complexity, which may affect real-time performance.

### 6.2. Future Work

- AI-generated content validation: Evaluate the framework on GenImage [5] and diffusion-generated forgery benchmarks (e.g., DIRE [19]) to address modern generative threats.
- Real CCTV dataset evaluation: Partner with surveillance system providers to test on authentic CCTV footage with verified tampering cases.
- Adaptive fusion mechanisms: Explore attention-based feature weighting for dynamic adjustment to different sensor types.
- Real-time optimization: Develop lightweight variants using knowledge distillation or neural architecture search for edge deployment.

## 7. Conclusions

In this work, we present MultiFusion, a proposed framework for image tampering detection that combines SRM-based noise residuals, local texture features extracted by EfficientNet-B0, and global structure features captured by a vision transformer. Traditional methods typically rely on a single source of features, whereas our model combines complementary information for enhanced robustness against varied manipulations, particularly those most relevant to surveillance systems, CCTV footage, and images from low-quality sensors. Furthermore, we have developed a unified interpretation method that leverages the combination of Grad-CAM from the convolutional stream with transformer attention to generate clear and informative visual explanations of manipulated regions. Through comprehensive experiments, we demonstrate that our approach achieves high detection accuracy on the CASIA 2.0 dataset while providing effective and interpretable visualizations, with significant performance gains compared to single-stream or single-feature models. These results support the proposed framework’s potential for deployment in security applications such as forensic analysis of sensor-captured imagery, real-time tampering detection in surveillance feeds, and authentication of visual data in IoT ecosystems. The MultiFusion framework proposed in this paper is very accurate and interpretable for general image forgery detection. Despite only being assessed on CASIA 2.0, its multimodal architecture is theoretically consistent with the needs of CCTV and sensor-based security, including noise, compression, and global consistency. This method has potential applications to video forensics, IoT authentication, and real-time tampering detection with future verification on actual surveillance data.

## Figures and Tables

**Table 1:** Summary of key recent works on image generation and forensic detection.

Ref.	Approach/Model	Dataset(s)	Key Findings/Contribution
[9]	Pseudo-numerical methods for diffusion models	Multiple	Enhanced stability and efficiency in diffusion sampling for high-dimensional spaces
[11]	Denoising Diffusion Implicit Models (DDIM)	Standard benchmarks	Faster sampling while maintaining high-quality image generation capabilities
[12]	Improved denoising diffusion probabilistic models	Various image datasets	Better variance schedules and architectural modifications for robust output
[15]	Latent Diffusion Models (LDM)	High-resolution datasets	Efficient high-resolution synthesis in compressed latent space with reduced computational cost
[16]	Text-to-image diffusion with deep language understanding	Text-image pairs	Photorealistic generation with improved semantic coherence across diverse prompts
[19]	DIRE for diffusion-generated image detection	Diffusion-generated images	Structural inconsistency analysis specific to diffusion-based synthesis artifacts
[20]	GenImage benchmark for AI-generated detection	Million-scale dataset	Comprehensive evaluation framework across multiple generative models and manipulation types
[21]	Vision transformer for forgery detection	Standard forensic datasets	Global inconsistency capture through transformer attention mechanisms

**Table 2:** Summary of the CASIA 2.0 dataset for image forgery detection.

Category	Image Count	Ground Truth	Description
Authentic (Au)	7491	Not Used	Original authentic images without any manipulation
Tampered (Tp)	5123	Not Used	Manipulated or forged images with various tampering operations
Total	12,614	-	Complete dataset comprising both authentic and tampered images

**Table 3:** Complete training and model configuration for the MultiFusion framework.

Parameter Category	Configuration Value
**Input Specifications**	
Image size	224×224×3 (resized from 256×256)
Normalization	Mean = [0.485, 0.456, 0.406], Std = [0.229, 0.224, 0.225]
**Training Settings**	
Batch size	16
Total epochs	50
Early stopping patience	10 epochs
Train/Val/Test split	70%/15%/15%
Random seed	42
**Optimization**	
Optimizer	Adam ($\beta_{1}=0.9$, $\beta_{2}=0.999$, $\epsilon =1\times 10^{-8}$)
Learning rate	$1\times 10^{-4}$ (Cosine annealing scheduler)
Weight decay	$1\times 10^{-4}$ (L2 regularization)
Loss function	Cross-entropy
**Regularization**	
Dropout rates	0.3 (first FC layer), 0.2 (second FC layer)
Data augmentation	Horizontal/vertical flip, rotation (±10°), brightness/contrast adjustment, Gaussian blur
**Model Architecture**	
CNN backbone	EfficientNet-B0
Vision transformer	ViT-Tiny (patch size 16×16, 12 layers, hidden size 192)
SRM configuration	Three fixed high-pass noise residual filters
Feature fusion	Concatenation (CNN: 512-dim, ViT: 256-dim, SRM: 64-dim → 832-dim total)
**Implementation Details**	
Framework	PyTorch 2.0.0, Python 3.9
Hardware	NVIDIA V100 (32 GB VRAM), 64 GB system RAM
Training time	∼6.5 h (50 epochs)
Inference latency	∼45 ms per image (batch size = 1)
**Reproducibility**	
Code availability	Available at: https://github.com/syedrizwanhassan/Tempered-image (accessed on 20 January 2026)
Dataset	CASIA 2.0 [22]
License	CC-BY 4.0

**Table 4:** Classification report on CASIA2 test set.

Class	Precision	Recall	F1-Score	Support
Authentic	0.9690	0.9645	0.9668	2985
Tampered	0.9648	0.9693	0.9671	3000
Accuracy	0.9669 (5985)			
Macro Avg	0.9669	0.9669	0.9669	5985
Weighted Avg	0.9669	0.9669	0.9669	5985

**Table 5:** Theoretical ablation study, showing the expected performance of different feature configurations.

Configuration	Exp. Acc. (%)	Exp. F1-Score	Exp. AUC	Primary Detection Capability
CNN-only	92.5 ± 1.2	0.920 ± 0.015	0.975 ± 0.010	Local texture and edge inconsistency
ViT-only	91.8 ± 1.5	0.915 ± 0.018	0.970 ± 0.012	Global structural and semantic inconsistency
SRM-only	85.0 ± 2.0	0.840 ± 0.025	0.920 ± 0.020	Noise residuals and compression artifacts
CNN + ViT	95.2 ± 0.8	0.950 ± 0.010	0.990 ± 0.005	Combined local and global structural analysis
Full MultiFusion	96.69	0.967	0.996	Comprehensive: texture + structure + noise

**Table 6:** Comparison of recent image forgery detection approaches (method categories).

Method Type	Preprocessing	Architecture	Explainability	Acc (%)	Ref.
Noise-aware Transformer	Denoising	ViT-based	Attention maps	96.1	[2]
CNN-Transformer Hybrid	Normalization	EfficientNet + ViT	Grad-CAM	96.7	[7]
SRM+CNN Fusion	SRM filtering	CNN-based	None	95.8	[1]
Diffusion-aware Detection	None	Custom CNN	Heatmaps	95.9	[19]
Proposed (MultiFusion)	DnCNN + SRM	CNN + ViT+ SRM	Grad-CAM + ViT	96.69	This

## Data Availability

The CASIA 2.0 dataset used in this study is publicly available at https://www.kaggle.com/datasets/sophatvathana/casia-dataset (accessed on 20 January 2026), and the code used is available at https://github.com/syedrizwanhassan/Tempered-image (accessed on 20 January 2026).
